# Bacterial Diversity Profiling around the Orca Seamount in the Bransfield Strait, Antarctica, Based on 16S rRNA Gene Amplicon Sequences

**DOI:** 10.1128/MRA.01290-20

**Published:** 2021-01-07

**Authors:** Wilbert Serrano, Raul M. Olaechea, Luis Cerpa, Jose Herrera, Aldo Indacochea

**Affiliations:** a Molecular Microbiology and Bacterial Genomics Laboratory, Universidad Científica del Sur, Lima, Perú; b Instituto Geológico Minero y Metalúrgico, Lima, Perú; c Carrera de Biología Marina, Universidad Científica del Sur, Lima, Perú; Indiana University, Bloomington

## Abstract

Hydrothermal vent activity is often associated with submarine volcanism. Here, we investigated the presence of microorganisms related to hydrothermal activity in the Orca seamount. Data profiling of the 16S rRNA gene amplicon sequences revealed a diversity pattern dominated mainly by the phyla *Proteobacteria*, *Acidobacteria*, *Planctomycetes*, and *Bacteroidetes*.

## ANNOUNCEMENT

Submerged seamounts in the oceans are generally vestiges of extinct volcanoes; these geological structures are produced by the lava that flows out from the internal part of the earth. The Orca seamount is located in the middle of the Bransfield Strait, a basin that extends along a passage between the South Shetland Island Arc and the Antarctic Peninsula (1). The Orca seamount is at present an inactive volcano; however, recently several studies have been conducted in order to detect some hydrothermal activity inside and around the crater (2). Due to its particular location, bordering a tectonic plate conjunction where trenches and fracture zones occur (3, 4), the Orca seamount may host several unknown deep sea hydrothermal vents. In this context, we report here a high-throughput 16S rRNA gene sequence analysis to study the microbiota in sediment samples collected near the Orca volcano crater.

Seven stations around the Orca seamount in the Bransfield Strait, Antarctica, were visited during the austral summer in February to March 2019 (Table 1). By means of a Van Veen sediment grab sampler, surface sediment samples were collected in 50-ml sterile Falcon tubes and immediately preserved at −20°C for later DNA analysis. DNA was extracted from 250- to 300-mg samples using a Soil DNA Isolation Plus kit (Norgen Biotek, Canada) according to the manufacturer’s protocol. The products of three independent extractions for each sample were pooled, and purified DNA was shipped to Macrogen, Inc. (Seoul, Republic of Korea) for PCR amplification and sequencing. PCR amplification of the V3/V4 hypervariable region was performed according to the Illumina 16S rRNA gene amplicon library method (5). Briefly, Illumina adapters were added to gene-specific primers 341F (CCTACGGGNGGCWGCAG) and 805R (GACTACHVGGGTATCTAATCC), and then a first PCR step was performed under thermocycler conditions according to reference 5. Further, sequencing libraries were constructed by index PCR using the Nextera XT Index kit, and paired-end sequencing was conducted using an Illumina MiSeq instrument. Raw data with paired-end reads (301 bp long) produced by the system are shown in Table 1. Raw sequences were evaluated with the FastQC package v0.11.8 (6). Microbial community analysis was conducted using mothur v1.39.5 (7) as implemented within the Galaxy platform (www.usegalaxy.org). Standard operating procedures, including those for data preparation (demultiplexing/denoising), quality control, chimera filtering, sequence alignments, clustering, and sequence classification steps, were as described by Schloss et al. (7). The SILVA database v128 (8) was used to assign operational taxonomic units (OTUs) at 97% similarity. The VSEARCH tool (9) was used for chimera removal.

**TABLE 1:** Summary of sample collection data and accession numbers

Sample	Collection coordinates	Collection depth (m)	No. of reads before quality control	No. of reads after quality control	Recovery (%)	SRA accession no.
E1	62.43S, 58.52W	1,256	220,679	76,607	35	SRX9195869
E3	62.52S, 58.54W	1,640	280,924	84,977	30	SRX9195870
E4	62.52S, 58.44W	1,636	256,160	74,372	29	SRX9195871
E5	62.47S, 58.32W	1,565	252,428	74,419	29	SRX9195872
E6	62.43S, 58.40W	1,090	212,316	79,467	37	SRX9195873
E7	62.42S, 58.3W	1,605	263,490	79,312	30	SRX9195874
E8	62.38S, 58.40W	1,400	237,638	72,011	30	SRX9195875

**FIG 1:**
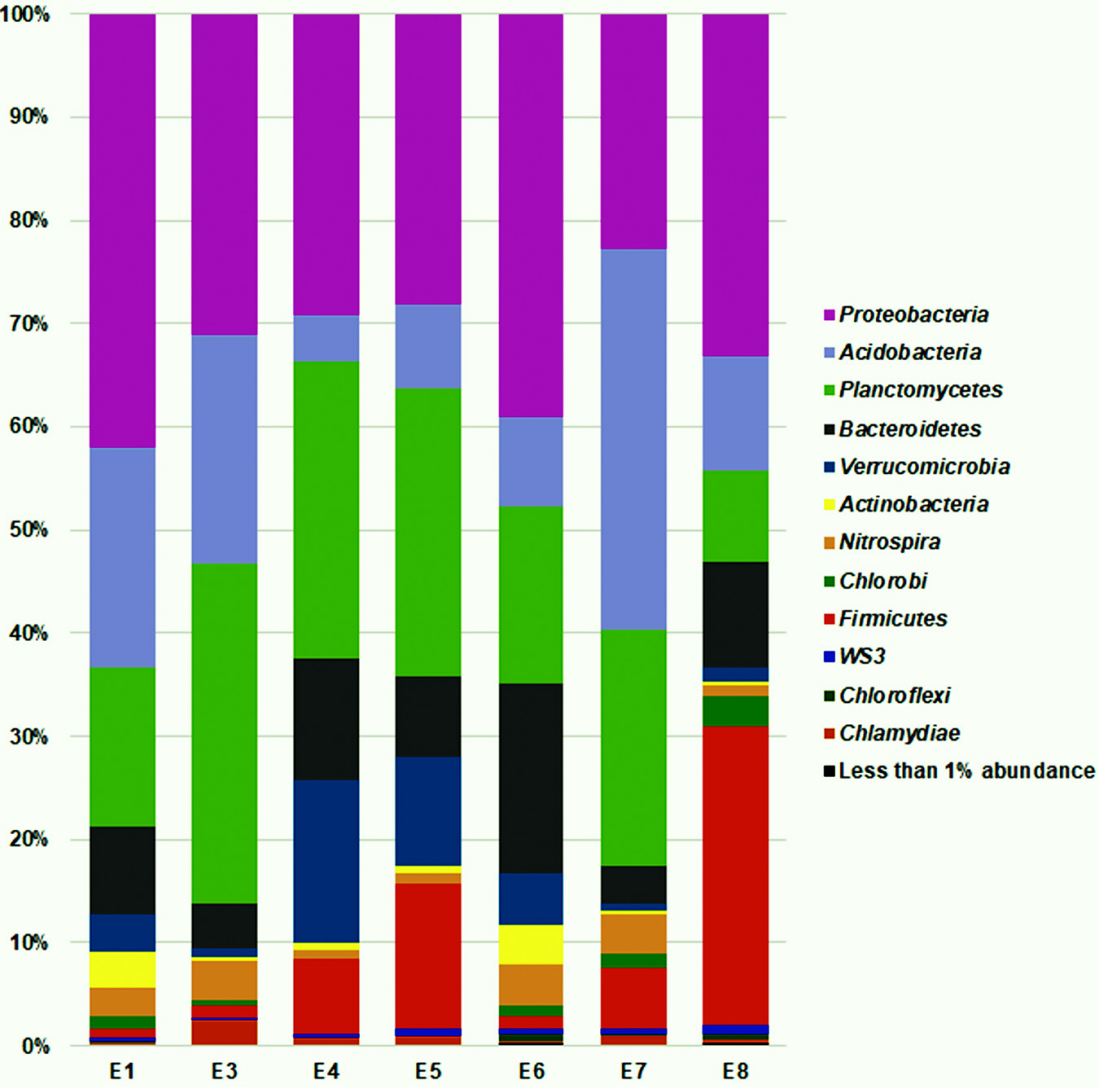
Bar chart representing bacterial diversity at seven stations around the Orca seamount volcano, based on 16S rRNA amplicon analysis. Relative abundances at the phylum level are shown.

Taxonomic classification at the phylum level (Fig. 1) showed that the bacterial taxa were almost the same for all analyzed samples, differing mainly in abundances. The dominant phyla were *Proteobacteria* (22.7 to 41.8%), *Acidobacteria* (4.4 to 36.8%), *Planctomycetes* (8.7 to 32.8%), and *Bacteroidetes* (3.8 to 18.2%). It is important to remark here that, within the *Proteobacteria*, members of the classes *Epsilonproteobacteria* and *Zetaproteobacteria* were present only at stations E1, E6, and E8. The number of *Zetaproteobacteria* OTUs was 3-fold higher at station E8. It is noteworthy that the first described and validly named member of the class *Zetaproteobacteria*, the species Mariprofundus ferrooxydans, was isolated from Fe-rich microbial mats associated with Fe(II)-rich fluids from seamount hydrothermal vents (10).

### Data availability.

The 16S rRNA gene amplicon raw read data set was deposited in GenBank under the SRA accession numbers SRX9195869 (E1), SRX9195870 (E3), SRX9195871 (E4), SRX9195872 (E5), SRX9195873 (E6), SRX9195874 (E7), and SRX9195875 (E8). The associated BioProject accession number is PRJNA610614.
